# Pediatric Pisiform Dislocation: A Case Report

**DOI:** 10.5811/cpcem.47400

**Published:** 2025-11-16

**Authors:** John Wahhab, Iswarya Vimalan Jeya, Jackson R. Huttner

**Affiliations:** Chicago Medical School, Rosalind Franklin University of Medicine and Science, North Chicago, Illinois

**Keywords:** pisiform bone, pisiform dislocation, pediatrics, wrist injuries, case report

## Abstract

**Introduction:**

Dislocations of the pisiform bone are rare, and literature on this injury is sparse. The uncommon nature of this condition, as well as limited data, makes recognition and diagnosis difficult, increasing the chances these injuries may be overlooked. Missing this diagnosis can lead to pain, reduced joint function, and nerve damage.

**Case Report:**

We present a case of pediatric pisiform dislocation and discuss the diagnosis and treatment in an emergency department setting.

**Conclusion:**

Prompt diagnosis and treatment of pisiform dislocations are vital to ensure favorable outcomes.

## INTRODUCTION

The pisiform bone is a carpal bone of the wrist within the flexor carpi ulnaris tendon, which functions to enhance flexion of the joint. 1,2 Pisiform dislocations are rare, and there is little documentation in the literature. Diagnosing pisiform dislocations at an early stage can be difficult. 1 These injuries often may go unnoticed, the consequence of which can result in significant pain for the patient and impairment of joint functionality. 3,4 Cases in the current literature describe primarily adult patients. 5 In this report we describe the diagnosis and treatment of a pediatric patient with a pisiform dislocation and a concomitant distal radius fracture, with the goal of highlighting the considerations when managing this rare condition.

## CASE REPORT

An 11-year-old, right-handed boy presented to the emergency department (ED) after injury to his right wrist. He reported that he was at football practice doing a blocking drill when his teammate’s facemask hit his wrist. Emergency medical services responders placed a temporary splint and brought him to the ED. He complained of a sharp, stabbing pain in his wrist that was worse with movement and better with rest. Initial vital signs were temperature 98.7 °F; heart rate 85 beats per minute; respiratory rate 18 breaths per minute; oxygen saturation 98% on room air; and blood pressure 120/67 millimeters of mercury. His physical exam revealed swelling, deformity, and tenderness of the right wrist, with light touch sensation intact distal to the injury. Range of motion of the wrist was decreased secondary to pain; there were no other signs of trauma on physical exam.

**Image 1:**
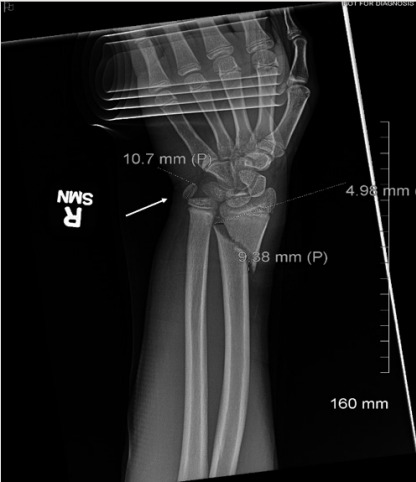
Right wrist radiograph, anteroposterior view. Arrow indicates the dislocated pisiform.

**Image 2:**
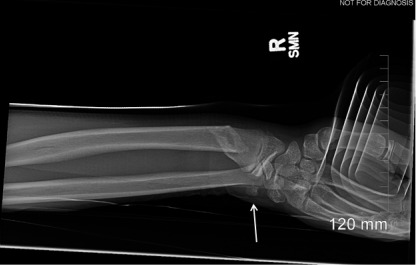
Right wrist radiograph, oblique view, of a child with pisiform bone dislocation. Arrow indicates the dislocated pisiform.

Radiographs of the right wrist were performed and showed an oblique fracture of the distal radial metadiaphysis with subcentimeter shortening and subcentimeter radial-sided displacement as well as mild anterior apex angulation. In addition, there was an ovoid, well-corticated ossific density that was suspicious for pisiform dislocation (Images 1 and 2). We obtained only two radiographic views, anterior-posterior and oblique. A lateral view could not be taken because of the patient’s inability to tolerate the imaging procedure due to pain.

Orthopedic consult did not recommend an attempt at closed reduction, instead recommending that he be transferred to a children’s hospital for urgent closed reduction. At the accepting hospital, pediatric orthopedic surgery was consulted. An attempt to reduce the pisiform dislocation by closed reduction was unsuccessful. He was placed in a splint and discharged. The patient was asked to follow up in the hand surgery clinic; however, he was lost to follow-up.

## DISCUSSION

The injury is described as a rupture of the anchoring ligaments of the pisiform, due to the powerful contraction of the flexor carpi ulnaris tendon during forced extension of the wrist. 1 Pisiform dislocations are considered uncommon, with a lack of agreement on the preferred treatment method. In acute cases, pisiform dislocation injuries are addressed with closed reduction and immobilization in an attempt to stabilize the injury non-surgically through the use of external support such as a plaster cast. 6,7 Open reduction is only recommended when there are signs of neurological injury to the ulnar nerve, or when the patient has sustained serious crush injuries, or after the failure of attempted closed reduction. 5,8 Open reduction is often accompanied by pinning or primary excision of the pisiform bone. 4 Surgical removal of the pisiform offers better clinical outcomes due to a faster recovery and restoration of original function. 6 A high incidence of pisiform instability and repeated dislocations have been reported after initial intervention, and they respond poorly to both open and closed reduction. 4

Most cases in the literature involve adult patients and describe dislocations in the anterior and posterior directions, while distal dislocations are rarer. 6,9,10 Our case differs from those reported, as it encompasses a pisiform dislocation in a pediatric patient,. 5,7,9 Moreover, our patient had a concomitant oblique fracture of the distal radial metadiaphysis. The immediate identification and diagnosis of this pisiform dislocation in the ED should be noted, as it generated a focused treatment plan.

*CPC-EM Capsule*

What do we already know about this clinical entity?

*Reports of pisiform bone dislocation are sparse, leading to significant debate regarding optimal treatment.*

What makes this presentation of disease reportable?

*We report a case of pisiform dislocation in a child with a concomitant distal radius fracture.*

What is the major learning point?

*Pisiform dislocations are frequently missed. Our goal was to shed light on this injury and how to diagnose and treat it.*

How might this improve emergency medicine practice?

*Timely management of pisiform dislocations is crucial to prevent chronic pain, disability, and nerve damage.*

Pisiform dislocation injuries are reported to be misdiagnosed in emergency settings 4 due to their infrequency and unfamiliar appearance on radiograph. 1 The injury is often recognized only after prolonged wrist pain when the patient is directed to a specialist. 4 In instances of delayed diagnosis, a direct excision of the pisiform is recommended. 4 Further research and reporting of the results of different treatment options may improve diagnosis and reduce patient morbidity.

In this case report, we sought to address the management of this rare injury in a pediatric patient, to bring attention to the consideration of pisiform dislocation in hand and wrist trauma, and to emphasize the need for urgent treatment. Our initial management was conservative, based on the existing literature. 6,7 We were fortunate to be able to make an expedient diagnosis and intervention, thus reducing the risk of pathological development of the joint. Identifying and managing the injury at an earlier stage can help avoid potential complications such as recurrence and instability of the pisiform bone. 6,9,10 Missed diagnoses can contribute to difficulty associated with chronic wrist pain, ulnar nerve injury, and post-traumatic arthritis.^11^ Recurrent dislocations can result in neurological damage to the ulnar nerve and warrant an emergency reduction; such neurological injury may be avoided through a proper initial assessment. 8

## CONCLUSION

It is critical that all physicians be able to accurately diagnose and properly treat pisiform dislocations. Dislocation of the pisiform bone is a rare type of orthopedic injury, especially among the pediatric population, making it a frequently missed diagnosis. A missed or delayed diagnosis can lead to recurrent pain, decreased range of motion and functionality of the affected hand, permanent nerve damage, and need for surgical intervention. Conservative management including non-surgical intervention and closed reduction are recommended. Surgery should be reserved for cases that involve injury to the ulnar nerve or for chronic dislocations. Our report reveals the importance of prompt diagnosis and appropriate treatment of pediatric pisiform dislocations, thereby ensuring the most favorable patient outcomes and prevention of further complications.
